# A Hazard *in Utero*?

**Published:** 2004-11

**Authors:** Julian Josephson

## Bisphenol A More Potent than Expected

Environmental estrogens are a structurally diverse group of chemicals that partially mimic the effects of endogenous estrogens. Scientists believe the wide use of environmental estrogens such as bisphenol A (BPA), a component of epoxy resins and polycarbonate plastics, may help explain the rising incidence of birth defects and certain cancers. It is further believed that the developing embryo is more vulnerable to the effects of environmental estrogens than adult animals, but until now it has been difficult to determine these effects directly in embryos. In this issue, Josephine G. Lemmen of the Netherlands Institute for Developmental Biology and colleagues investigate the use of a new transgenic mouse model to study such effects **[EHP** **112:1544–1549]**.

During the late 1990s, it was first suggested that prenatal exposure to BPA might cause reproductive abnormalities; experimental data, however, led to contradictory findings. For instance, one 1999 study showed prostate enlargement in offspring of BPA-exposed mice, but other studies reported no effect. Although BPA concentrations in amniotic fluid at 15–18 weeks’ gestation have been shown to be five times those in the serum of pregnant and nonpregnant women—suggesting possible accumulation in the embryo—this could not be confirmed through animal experiments.

Lemmen and her colleagues recently developed a transgenic mouse in which estrogen-responsive elements are coupled with the reporter enzyme luciferase. Direct activation of estrogenic receptors (ERs) is detected photometrically by measuring luciferase activity, which allows quantitative and time-course analysis of target gene activation *in vivo*. The C57Bl/6J mouse strain used in this model had previously been shown to be especially sensitive to the effects of estrogen exposure.

This model was used to evaluate the ability of BPA to activate endogenous ERs in mouse embryos, as compared with the strong estrogens diethylstilbestrol (DES) and 17β-estradiol dipropionate (EP). Exposure of pregnant mice to varying dosages of all three estrogens activated the endogenous ERs in their embryos. Exposure to DES and EP showed a dose- and time-dependent induction of luciferase activity. For all DES exposures, peak activity was seen at 8 hours after exposure. For EP, peak activity was seen at 24 hours after exposure.

Like DES, BPA showed a transient induction of luciferase activity. Surprisingly, though, BPA was found to be more potent *in vivo* than would have been expected on the basis of its activity *in vitro*. *In utero* luciferase activation by BPA in transgenic embryos at 8 hours after exposure was significant at a dosage as low as 1 milligram BPA per kilogram body weight, compared with controls.

One possible explanation for a higher potency *in utero* may be that *in vivo* BPA is converted to metabolites with enhanced estrogenicity, as some previous studies have suggested. Yet another explanation could be that BPA has a lower affinity with steroid-binding proteins present in serum, which gives it a greater bioavailability than, say, EP. However, these explanations cannot account for the *in vivo* versus *in vitro* potency of BPA as compared with DES, because neither has a high affinity to binding proteins.

Although BPA’s intrinsic activity is lower than that of DES or EP, it still was more potent *in vivo* than would be estimated from *in vitro* assays. Effects on individual embryonic organs have not yet been evaluated and could possibly provide even more sensitive end points than whole embryos. Although the Lemmen study model showed that the effects of BPA did not persist like those of the other estrogens, its biological effects in exposed embryos should continue to be assessed, perhaps with other types of models.

